# Effect of Cigarette Smoke on Salivary Total Antioxidant Capacity

**DOI:** 10.15171/joddd.2015.049

**Published:** 2015-12-30

**Authors:** Sedigheh Bakhtiari, Somayyeh Azimi, Masoumeh Mehdipour, Somayyeh Amini, Zahra Elmi, Zahra Namazi

**Affiliations:** ^1^Associate Professor, Department of Oral medicine, Shahid Beheshti University of Medical Sciences, Tehran, Iran; ^2^Assistant Professor, Department of Oral medicine, Shahid Beheshti University of Medical Sciences, Tehran, Iran; ^3^Honorary Research Fellow, University of Western Australia, Perth, Australia; ^4^Dentist, Private Practice, Tehran, Iran; ^5^Post-graduate Student, Department of Oral medicine, Shahid Beheshti University of Medical Sciences, Tehran, Iran; ^6^PHD Student, Department of Dental Materials, Tehran University of Medical Sciences, Tehran, Iran

**Keywords:** Antioxidant, saliva, smoking

## Abstract

***Background and aims.*** Cigarette smoke can induce oral cancer by its free radicals and oxidative damage. Salivary anti-oxidants system is believed to have an important role in defense mechanisms against oxidative stress. This study was compared total antioxidant capacity (TAoC) of saliva in smokers and nonsmokers.

***Materials and methods***. In this cross-sectional study, 30 male smokers with mean age of 45.23 years and 30 nonsmokers with mean age of 45.30 years participated. Unstimulated whole saliva samples were collected in the morning in two groups by spitting method. TAoC of saliva was measured with the special kit in two groups at the same time. Statistical analysis was performed by covariance test.

***Results.*** The mean salivary TAoC in nonsmokers (0.741±0.123 U/ml) was higher than that in smokers (0.529±0.167 U/ml). This difference was statistically significant (P<0.001).

***Conclusion.*** Smoking can alter salivary antioxidant capacity.

## Introduction

 Cigarette smoke comprises several materials including carbon monoxide, nitrogen, nicotine, and free radicals such as superoxide, hydroxyl, hydrogen peroxide and reactive oxygen (O^2^-). These compounds are associated with increased incidence of cancer in different parts of the body including the oral cavity.^1-4^ Saliva is the first body liquid to come in contact with foreign substances or gases such as the cigarette smoke.^5^The saliva contains immunoglobulin and enzymes such as lactoferrin, lysozyme, and histamine. They play a crucial role in defense mechanisms against free radicals and thereby oral cancer occurrence.^6-9^

 Antioxidants protect body against free radicals.^10^Salivary antioxidants is composed of molecules such as uric acid, glutathione, and enzymes,^8^ including superoxide dismutase (SOD), catalase (CAT) peroxidase (POX) and glutathione peroxidase (GSH-PX).^11^ Cigarette smoke may attack antioxidant system. Salivary antioxidants activity in smokers may be not protective against cumulative stressors. The salivary antioxidant system has been drawing increased attention in recent years. Numerous studies have shown changes in activity of salivary antioxidants system in smokers and in patients with squamous cell carcinoma (SCC) comparing to control group, but there are some differences between them.^11-16^

 In previous study salivary superoxide dismutase activity in smokers was compared with non-smokers, and results showed that the mean value of superoxide dismutase activity was significantly higher in the smoking group, while no detectable activity level was found in nonsmokers.^14^ This study was performed to investigate the effect of smoking on salivary total antioxidant capacity (TAoC).

## Materials and Methods

 In this cross-sectional analytical study, 30 male smokers (with the history of 5 or more packs/year of smoking) and 30 non-smokers were selected by simple non-randomized sampling method from patients attending the dental school of Shahid Beheshti University of Medical Sciences, Tehran, Iran. The study population were generally healthy and had not been taking any medications for systemic diseases, in at least previous 3 months. Informed consents were taken from patients. The study approved by the ethics committee of Shahid Beheshti University of Medical Sciences (approval code: 145).

 The participants were requested not eat or drink two hours prior to saliva collection. The smokers were also prohibited from smoking for one hour before saliva collection. In order to gather whole unstimulated saliva, the patients were asked to spit their saliva into 50 ml falcon tubes. Collection of saliva was done in an upright position, between 9 and 12 o’clock in the morning. To separate squamous cells and derbies, samples were centrifuged (5000g at 4°C) immediately for 15 minutes (Hetich, Germany).The centrifuged samples were preserved at −70°Cto be assessed later.

 Total antioxidant capacity (TAoC) was evaluated using antioxidant assay kit (Cayman Chemical, Cat No.709001, USA).^4^

 Each sample was assessed two times by an experienced technician who was blinded to the cases. We used ELISA Reader machine (Anthoos 2020, Germany) to read the results. SPSS 16 software was used for statistical analyzing.

## Results

 In this study, 30 smoker men (mean age, 45.23 ± 12.27) with a history of smoking a mean number of16.5 ± 11.32 packs/year (minimum, 5; maximum, 40) and 30 non-smoker men (mean age, 45.30 ± 12.34) were examined. Covariance test showed that age had no effect on the TAoC (P = 0.614).

 The findings revealed a significantly higher salivary TAoC in nonsmokers (0.741± 0.123 U/ml) than in smokers (0.529 ± 0.167 U/ml; P < 0.001; Table 1).

**Table 1:** Statistical index of salivary total antioxidant capacity (TAoC) in smokers and non-smokers

**Group**	**Number**	**Min**	**Max**	**Mean**	**SD**	**SE**
**Smokers**	30	0.365	1.008	0.529	0.167	0.030
**Non-smokers**	30	0.423	0.726	0.741	0.123	0.022

## Discussion

 This study compared total antioxidant capacity of saliva in smokers and nonsmokers. The results showed the mean salivary total antioxidant capacity (TAoC) in nonsmokers was higher than smokers; and this difference was statistically significant (P <0.001). The result of the present study is in line with that of Ziborro and Bartosz.^13^

 Hammo Mahmoud et al^2^ measured plasma level of TAoC in smokers’ antecubital venous blood using a similar test to that of the present study and showed a significant reduction in total antioxidant status in smokers.

 Abdolsamadi et al^17^ also suggested cigarette smoking is related with a significant decrease in salivary antioxidant concentrations. In their study, the mean levels of salivary superoxide dismutase, glutathione peroxidase, and peroxidase were significantly lower in smokers.^17^ They concluded that the measurement of antioxidant agents in human saliva could be beneficial for estimating the level of oxidative stress caused by cigarette smoke.^17^

 It seems that low antioxidant values in smokers due to the presence of a high amount of free radicals in cigarette smoke generatingan oxidative stress in the smoker bodies causing an exhaustion of the antioxidants of the body.

 Baharvand et al^14^ demonstrated SOD activity in smokers and concluded that salivary SOD activity were significantly higher in smokers compared to nonsmokers.

 Saggu et al^18^ analyzed unstimulated saliva of 100 smokers by measuring the activity of salivary SOD and glutathione peroxidase and showed the a significantly higher mean value of SOD activity in the smokers’ group, while the levels of GSH-Px activity were significantly higher in the nonsmoking group. They suggested these measurements could be helpful for determining the level of oxidative stress caused by cigarette smoke and may help in patient’s education regarding the harmful effects of smoking and estimating the evolution of various oral diseases.^18^

 The measurement of total antioxidant activity, as performed in the present study, is better than measuring the fractional antioxidant, because the measurement of all known antioxidants is time consuming, many antioxidants may remain undiscovered, and the total activity may be greater than the sum of the individual antioxidants because of their cooperative interactions.^4^

 In contrast to the findings of the present study, Nagler et al^19^ showed that total salivary anti-oxidant capacity was significantly higher in smokers compared to nonsmokers.^19^

 The conflicting results between the studies may have arisen from differences in smoking patterns, the number and the age of the subjects, the type of tobacco, the cigarette design including filtration, paper and additives, and the method of antioxidant measurement.

 Fruits and vegetables have a considerable amount of antioxidants and the dietary habits of the studied population may affect the results as a confounding factor. Although, many researchers have evaluated antioxidants activity without evaluating the nutritional habits,^13,14^ this could be recommended for future studies. In the present study, all of the participants were selected from a single dental clinic, and it might be safe to say that they were from the same socio-economic class with probably same nutritional habits.

 Although it could be stated that tobacco smoke can alter the antioxidative capability of saliva, there is controversy over the exact cause of these changes between the studies.^17-21^Considering the importance of salivary antioxidant profile analysis for understanding the relation between saliva and free radicals, more studies are warranted in this field.

## Conclusion

 The present study demonstrated that cigarette smoking leads to alternation of salivary TAoC. Being protective against oxidative damage in oral tissues, the use of antioxidant agents might boost the salivary antioxidant system and help in decreasing the incidence of oral cancers among individuals with smoking habits.

## Acknowledgements

 This paper was written based on the undergraduate thesis by Dr. Amini, which was successfully completed in cooperation with the Department of Oral Medicine at Shahid Beheshti University of Medical Sciences Dental School. Dr. Azimi acknowledges the support of postgraduate award from University of Western Australia.
